# Genomic landscape and efficacy of HER2-targeted therapy in patients with *HER2*-mutant non-small cell lung cancer

**DOI:** 10.3389/fonc.2023.1121708

**Published:** 2023-04-03

**Authors:** Yanjie Han, Yuanyuan Xiong, Tao Lu, Rongrong Chen, Yuan Liu, Hui Tang, Ruixuan Geng, Yingyi Wang

**Affiliations:** ^1^ Department of Medical Oncology, Peking Union Medical College Hospital, Chinese Academy of Medical Sciences and Peking Union Medical College, Beijing, China; ^2^ 4 + 4 Medical Doctor (MD) Program, Chinese Academy of Medical Sciences and Peking Union Medical College, Beijing, China; ^3^ Geneplus-Beijing, Beijing, China; ^4^ Molecular Pathology Research Center, Department of Pathology, Peking Union Medical College Hospital, and Chinese Academy of Medical Sciences and Peking Union Medical College, Beijing, China; ^5^ Department of International Medical Services, Peking Union Medical College Hospital, Chinese Academy of Medical Sciences and Peking Union Medical College, Beijing, China

**Keywords:** *HER2*, non-small cell lung cancer, HER2-TKI, resistance mechanism, targeted sequencing

## Abstract

**Background:**

HER2-targeted therapy provides survival benefits to *HER2*-mutant non-small cell lung cancer (NSCLC). A better understanding of the clinical and genomic characterization of treatment-naïve *HER2*-positive NSCLC, as well as the efficacy of and resistance to HER2-targeted therapy in *HER2*-altered NSCLC, could promote further improvement of HER2 targeted therapy.

**Methods:**

*HER2*-altered NSCLC patients was retrospectively included and their genomic profiles were performed by next-generation sequencing. The clinical outcomes included overall response rate, disease control rate and progression-free survival.

**Results:**

Among 176 treatment-naïve patients with *HER2* alterations, 64.8% harbored *HER2* mutations with/without *HER2* amplification, and 35.2% carried *HER2* amplification only. Molecular characterization was correlated with tumor stage that late-stage NSCLC with *HER2* oncogenic mutations showed a higher prevalence of *TP53* mutations and a higher tumor mutation burden. However, this correlation was not found in patients with *HER2* amplification only. Twenty-one patients with *HER2* alterations treated with pyrotinib or afatinib were retrospectively enrolled. Pyrotinib yielded a longer median progression-free survival than afatinib (5.9 [95% CI, 3.8-13.0] vs. 4.0 months [95% CI, 1.9-6.3], *P* = 0.06) in these patients. Analysis of the genomic profiles before and after anti-HER2 targeted therapies identified *de novo HER2* copy number gain and G518W mutation, as well as mutations involving DNA damage repair signaling, SWI–SNF complex, and epigenetic regulations as potential resistance mechanisms.

**Conclusion:**

*HER2*-mutant NSCLC had different molecular features from *HER2*-amplified NSCLC, and its genomic profile was dependent of tumor stage. Pyrotinib had superior therapeutic effects than afatinib in *HER2*-altered NSCLC, although larger cohorts are warranted to validate it. *HER2*-dependent and -independent resistance mechanisms to afatinib and pyrotinib were unveiled.

## 1 Introduction

Human epidermal growth factor receptor 2 (HER2), a member of the ERBB family of tyrosine kinase receptors, has been suggested to be a therapeutic target of non-small cell lung cancer (NSCLC) in recent years. Its oncogenic mutations occur in 2-4% of NSCLC and are almost mutually exclusive with activating mutations in other oncogenic drivers. Patients with *HER2* mutations were common in women, nonsmokers, and adenocarcinoma (1–4). Although exon 20 insertions are the most common mutations and emerging as a clinically validated therapeutic target, point mutations and other insertions have also been suggested to be actionable (5–7). The heterogeneity of *HER2* mutations offers therapeutic challenges. Compared with *HER2* mutations, *HER2* amplification has been reported as a distinct entity and therapeutic target that might be less relevant in lung carcinogenesis (1, 8). However, the co-alteration profiles of *HER2* mutations and amplification in treatment-naïve NSCLC remain largely unknown.

Molecular testing based on next-generation sequencing (NGS) is not routinely conducted for early-stage NSCLC experiencing curative-intent pulmonary resection in many medical centers. However, early-stage lung cancers have a high risk of recurrence, with 5-year disease-free survival ranging from 36% to 82% (9). In the event of recurrent disease, tumor tissue samples for molecular testing are unavailable in many cases. The absence of molecular profiles brings challenges to better guide treatment strategy. Therefore, elucidating the molecular characteristics of *HER2* alterations, especially in treatment-naive NSCLC patients at different stages, is essential to refine the therapeutic strategy.

Recently, several studies supported the clinical response of anti-*HER2* drugs in *HER2*-positive breast cancer, gastric adenocarcinoma, and gastroesophageal adenocarcinoma, which promotes the exploration of anti-*HER2* agents in NSCLC. Pan-HER tyrosine kinase inhibitors (TKIs) such as afatinib, pyrotinib, dacomitinib, neratinib, and poziotinib have been evaluated in *HER2*-positive NSCLC patients, with several of them showing effectiveness (10, 11). Afatinib is a selective inhibitor of the ERBB protein family, which irreversibly blocks the signaling of these proteins (12). Several studies have reported the clinical response in *HER2*-positive NSCLC patients with an objective response rate (ORR) of 7.7-33% and a median progression-free survival (mPFS) of 3.2-3.7 months (11, 13). However, increasing evidence suggests the disappointing effectiveness of afatinib in *HER2*-mutated NSCLC. In a phase II trial that enrolled eighteen patients with *HER2*-positive advanced NSCLC, no patient achieved an objective response and harbored a mPFS of 2.76 months (14). Xu et al. reported that chemotherapy could benefit more than afatinib to *HER2-*mutated advanced lung cancer, especially in those with A775_G776insYVMA (15). Therefore, the clinical application of afatinib in *HER2*-positive NSCLC needs further exploration. Pyrotinib, another irreversible pan-HER TKI, has been suggested to be a beneficial therapeutic agent in *HER2*-mutated NSCLC (6, 16). In patient-derived organoids and xenografts harboring *HER2* A775_G776YVMA advanced NSCLC, pyrotinib showed a superior antitumor activity than afatinib or T-DM1 (17). Several studies demonstrated the clinical response of pyrotinib in *HER2*-positive metastasis NSCLC with an ORR of 19.2-53.3% and a mPFS of 5.6-6.8 months (6, 11). Although afatinib and pyrotinib offer clinical benefits for *HER2*-mutated NSCLC, resistance to targeted therapy is observed in the clinical setting and brings a significant challenge in cancer management. Uncovering the mechanism underlying the acquisition of *HER2* TKI resistance is warranted to overcome the resistance.

In this retrospective study, we aimed to dissect the molecular characteristics of *HER2* alterations in treatment-naïve NSCLC, especially the differences of molecular characteristics between early- and late-stage NSCLC with *HER2* alterations, assess the efficacy of afatinib and pyrotinib in *HER2*-positive NSCLC patients, and uncover the potential resistance mechanism to them.

## 2 Methods

### 2.1 Patients and cohort

A total of 176 treatment-naïve NSCLC patients with *HER2* alterations receiving commercial NGS of 1,021 cancer-related gene panel (Geneplus-Beijing, Beijing, China) between August 2016 and March 2022 were retrospectively enrolled. Twenty-one patients with *HER2* alterations who received targeted therapy at Peking Union Medical College Hospital between March 2018 and April 2022 were retrospectively enrolled to investigate the efficacy and resistance mechanism to anti-HER2 TKIs. Written informed consent was obtained from each patient. All procedures conformed to the principles of the Helsinki Declaration with approval by institutional review boards of Peking Union Medical College Hospital.

### 2.2 Data collection and response assessment

The clinicopathological data, including age, sex, tumor histology, smoking status, and treatment information, were collected from the patient medical record. The outcome measures are PFS, ORR and disease control rate (DCR) assessed by standard radiologic criteria. PFS was defined as the interval from initial anti-HER2 targeted therapy to progression or death from any cause. ORR was defined as the proportion of patients with a partial response (PR) or complete response (CR). DCR was defined as the proportion of patients with a stable disease or PR or CR.

### 2.3 DNA extraction and targeted next-generation sequencing

DNA was isolated from tissue samples, peripheral blood, cerebrospinal fluid and hydrothorax using commercial kits (Qiagen, Hilden, Germany). The KAPA Library Preparation Kit (Kapa Biosystems, Wilmington, MA, USA) was used to prepare indexed Illumina NGS libraries. Custom-designed 1,021 or 59 cancer-related gene panels were used to hybridize the DNA libraries, and their selected regions and genes are listed in **Supplementary Tables 1** and **2**. The hybridized libraries were sequenced using a 100-bp paired-end configuration on a DNBSEQ-T7RS sequencer (MGI Tech, Shenzhen, China).

After the removal of terminal adaptor sequences and low-quality reads with FASTP (18), the remaining reads were mapped to the reference human genome (hg19) and aligned using the Burrows-Wheel Aligner (version 0.7.12-r1039) with default parameters. GATK (3.4–46-gbc02625) and MuTect2 (1.1.4) were used to call somatic single nucleotide variants and small insertions and deletions. Contra (2.0.8) was used to identify copy number variations (19). NCsv (in-house algorithm 0.2.3) was employed to detect structural variants (20, 21). All candidate variants were manually confirmed by using the integrative genomics viewer browser. Variants were filtered to exclude clonal hematopoietic mutations with an inhouse database of clonal hematopoiesis variants of >10000 pan-cancer patients and healthy individuals (22), germline mutations in dbSNP, as well as variants that occur at a population frequency of >1% in the Exome Sequencing Project.

### 2.4 Tumor mutation burden (TMB) and PD-L1 expression evaluation

The TMB was determined as the number of somatic nonsynonymous single nucleotide variants and small insertions/deletions per mega-base in the coding region (with VAF ≥ 0.03) (23). Immunohistochemistry with the PD-L1 IHC 22C3 pharmDx assay (Agilent Technologies, Santa Clara, CA, USA) was performed to evaluate PD-L1 expression of tumor tissues.

### 2.5 Statistical analyses

Statistical analyses were performed using SPSS version 19.0 (SPSS Company, Chicago, IL). The Mann-Whiney U test and Student’s t-test were used for non-normally and normally distributed continuous variables, respectively. The comparison of categorical variables was conducted with Pearson’s χ2 test or Fisher’s exact tests. All statistical tests were performed with two-sided methods, and *P* < 0.05 was considered to indicate statistical significance.

## 3 Results

### 3.1 The molecular characteristics of treatment-naïve NSCLC patients with *HER2* alterations

A total of 176 treatment-naïve NSCLC patients with *HER2* alterations were enrolled to analyze the co-alteration profile of *HER2* alterations. The median age at diagnosis was 59.5 years old (range, 25-80), with 56.8% (100/176) males and 30.1% (53/176) smokers. Most patients had stage IV disease (54.0%, 95/176), followed by stage I (21.6%, 38/176), stage III (16.4%, 29/176), and stage II (8.0%, 14/176). *Via* clinicopathological examination, 86.4% (152/176) patients had adenocarcinoma, with 7.4% (13/176) squamous cell carcinoma, 1.7% (3/176) adenosquamous carcinoma and 4.5% (8/176) unclear tumor histology. Of all 176 tumor tissues used for 1,021 gene panel sequencing, 123 (69.9%) samples were from primary lesions, 33 (18.7%) from metastases lesions and 20 (11.4%) were of unknown origin (**Table 1**).

**Table 1:** Demographic and baseline characteristics of 176 treatment-naïve NSCLC patients with *HER2* alterations.

	n = 176
Age, years	
Mean (SD)	58.5 (11.0)
Median (min-max)	59.5 (25-80)
Gender, n (%)	
Male	100 (56.8)
Female	76 (43.2)
Smoking, n (%)	
Ever	53 (30.1)
Never	62 (35.2)
Unknown	61 (34.7)
Tumor stage, n (%)	
I	38 (21.6)
II	14 (8.0)
III	29 (16.4)
IV	95 (54.0)
Histology, n (%)	
Adenocarcinoma	152 (86.4)
Squamous cell carcinoma	13 (7.4)
Adenosquamous carcinoma	3 (1.7)
Unknown	8 (4.5)
Tumor site, n (%)	
Primary lesion	123 (69.9)
Metastases lesion	33 (18.7)
Unknown	20 (11.4)

**Figure 1:**
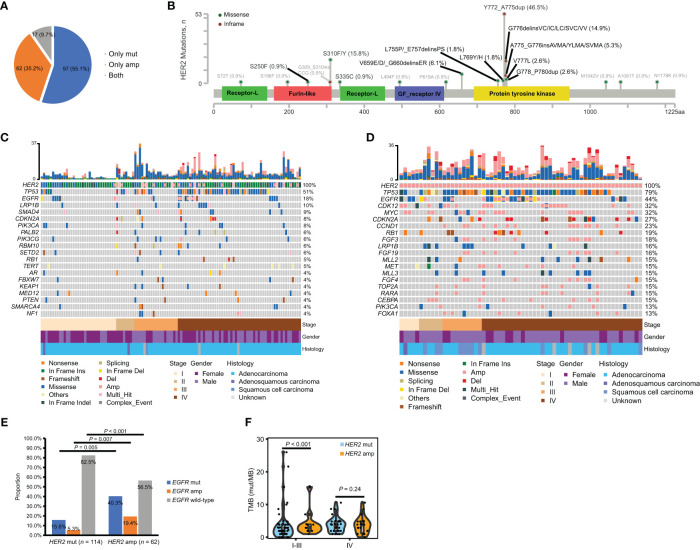
Incidence and co-mutation profiles among treatment-naïve *HER2*-altered NSCLC patients from the geneplus dataset. **(A)** Pie plot showing the incidence of *HER2* mutation only (*n* = 97), *HER2* amplification only (*n* = 62) or both *HER2* mutation and amplification (*n* = 17) within our cohort. **(B)** Oncogenic (black) and non-oncogenic mutations (gray) in *HER2*-mutant patients were localized to HER2 protein. **(C, D)** Heatmap displaying the mutation landscapes of patients with *HER2* mutations (*n* = 114) **(C)** and with *HER2* amplification only (*n* = 62) **(D)**. Each column represents data from a single patient. The number of somatic mutations of each patient and the mutation frequency of each gene are shown in the top and right respectively. The bottom heatmaps indicate key patient characteristics. **(E)** The proportion of *EGFR* mutation, amplification and *EGFR* wild-type in patients with *HER2* mutations (*n* = 114) and with *HER2* amplification only (*n* = 62). **(F)** Difference of TMB between patients with *HER2* mutations (*n* = 106) and with *HER2* amplification only (*n* = 60) in early- and late-stage disease respectively. Amp, amplification; mut, mutation; TMB, tumor mutation burden.

According to the sequencing data, a total of 1,945 somatic alterations were identified, with a mean of 11.1 alterations per sample. Among these alterations, 1,361 were substitution/small insertions and deletions, with 514 gene amplifications, 65 truncations, and 5 fusion/rearrangements. *HER2* mutations were detected in 114 (64.8%) patients, including 17 patients with *HER2* co-amplification. *HER2* amplification only was found in 62 (35.2%) patients (**Figure 1A**). A total of 32 types of *HER2* mutations were identified, including 22 (68.6%) oncogenic types, which are documented as pathogenic in the COSMIC database (**Figure 1B**). The tyrosine kinase domain (TKD) of *HER2* was detected as the most frequently mutated region (75.4%), followed by the furin-like domain (19.3%) and others (13.1%). Across all *HER2* oncogenic mutations, Y772_A775dup was the most common mutation (46.5%), followed by S310F/Y (15.8%) and G776delinsVC/IC/LC/SVC/VV (14.9%) (**Figure 1B**). A comparison of the clinical features between oncogenic TKD and non-TKD mutations indicated that patients with oncogenic non-TKD mutations were older than those with oncogenic TKD mutations (**Table 2**). Furthermore, patients with oncogenic non-TKD mutations had a significantly higher prevalence of *EGFR*, *KEAP1*, and *RBM10* mutations and *EGFR* amplification than those with oncogenic TKD mutations (*EGFR* mutation: 51.9% vs. 2.4%, *P* < 0.001; *KEAP1* mutation: 14.8% vs. 1.2%, *P* = 0.01; *RBM10* mutation: 18.5% vs. 0.0%, *P* = 0.001; *EGFR* amplification: 11.1 vs. 1.2%, *P* = 0.04).

**Table 2:** Baseline clinical and molecular characteristics between treatment-naïve patients with *HER2* oncogenic TKD and non-TKD mutations.

	TKD (*n* = 85)	non-TKD (*n* = 27)	*Pval*
Age, years			0.03
Mean (SD)	55.0 (11.7)	60.8 (10.2)	
Median (min-max)	55 (25-76)	63 (39-77)	
Gender, *n* (%)			0.18
Male	42 (49.4)	9 (33.3)	
Female	43 (50.6)	18 (66.7)	
Smoking, *n* (%)			0.4
Ever	20 (23.5)	5 (18.5)	
Never	29 (34.1)	13 (48.2)	
Unknown	36 (42.4)	9 (33.3)	
Histology, *n* (%)			0.56^a^
Adenocarcinoma	81 (95.3)	25 (92.6)	
Squamous cell carcinoma	1 (1.2)	0	
Adenosquamous carcinoma	1 (1.2)	1 (3.7)	
Unknown	2 (2.3)	1 (3.7)	
Tumor stage, *n* (%)			1.0^b^
I	23 (27.1)	10 (37.0)	
II	6 (7.0)	1 (3.7)	
III	16 (18.8)	3 (11.1)	
IV	40 (47.1)	13 (48.2)	
TMB test, *n* (%)			0.14
Yes	78 (91.8)	26 (96.3)	
No	7 (8.2)	1 (3.7)	
Mean (SD)	4.3 (4.8)	6.0 (5.7)	
Median (min-max)	2.9 (0-28.8)	4.0 (0-26.0)	
PD-L1 test, *n* (%)			0.37
Yes	19 (22.4)	7 (25.9)	
No	66 (77.6)	20 (74.1)	
TPS<1%	13 (68.4)	3 (42.9)	
TPS≥1%	6 (31.6)	4 (57.1)	

a Comparison between adenocarcinoma and non-adenocarcinoma.

b Comparison between I-III and IV.

Given the evidence that *HER2* mutations and amplification exhibit distinct entities in carcinogenesis (1, 8), we elucidated the co-alteration profiles of *HER2* mutations and amplification respectively. The most frequent concomitant alterations of *HER2* mutations with/without amplification were *TP53* (50.9%), *EGFR* (17.5%), *LRP1B* (9.6%), *SMAD4* (8.7%), *PIK3CA* (7.9%), *CDKN2A* (7.9%) and *SMARCA4* (7.0%) (**Figure 1C**). The most common concomitant alterations of *HER2* amplification only were *TP53* (79.0%), *EGFR* (43.6%), *CDK12* (32.3%), *MYC* (32.3%), *CDKN2A* (27.4%), *CCND1* (22.6%) and *RB1* (19.4%) (**Figure 1D**). Of note, patients with *HER2* amplification only had a significantly higher prevalence of *EGFR* mutation (40.3% vs. 15.8%, *P* = 0.005) and *EGFR* amplification (19.4% vs. 5.2%, *P* = 0.007), as well as lower prevalence of *EGFR* wild-type (56.5% vs. 82.5%, *P* < 0.001) than those with *HER2* mutations (**Figure 1E**). We further compared TMB between patients with *HER2* mutations and with *HER2* amplification only in early- and late-stage disease respectively. TMB data was available for 106 patients with *HER2* mutations and 60 patients with *HER2* amplification only. Our results showed that patients with *HER2* amplification only had a significantly higher TMB than those with *HER2* mutation in early-stage but not in late-stage disease (**Figure 1F**). Collectively, the molecular characteristics of *HER2* mutations and amplification differed.

### 3.2 The molecular characteristics of early- and late-stage NSCLC with *HER2* alterations

**Figure 2:**
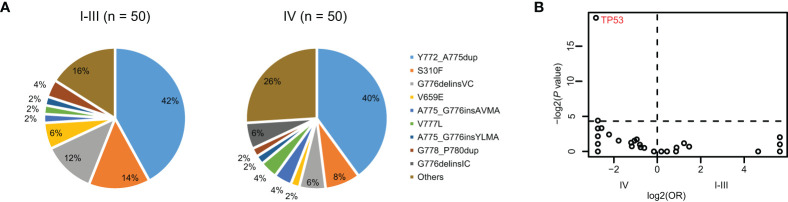
The differences of co-alteration profile between early- and late-stage NSCLC with *HER2* mutation. **(A)** Pie plot showing the incidence of *HER2* mutation types within early- (*n* = 50) and late-stage NSCLC (*n* = 50) with *HER2* mutation. **(B)** Volcano plots showing the difference of co-occurring alterations between early- and late-stage NSCLC with *HER2* mutation.

Among 114 patients with *HER2* mutations, 60 of them (52.6%) were early stage (stage I-III). Compared with late NSCLC, patients with early NSCLC were younger (**Supplementary Table 3**). To compare the mutational profiles between early- and late-stage NSCLC with *HER2* mutation and to avoid age as a potentially confounding variable, we conducted a propensity-matched comparison manually. As a result, a total of 100 matched patients were obtained, including 50 out of the 60 patients from the early-stage group and 50 out of 54 patients from the late-stage group. Their baseline characteristics are depicted in **Table 3**. The matched early- and late-stage patients had similar *HER2* mutation types (**Figure 2A**). Remarkably, the result of the co-alteration comparison between matched early- and late-stage patients indicated that *TP53* mutation was more frequently observed in patients with late-stage disease (78.0% vs. 30.0%, *P* < 0.001) (**Figure 2B**). Moreover, patients with late-stage NSCLC had significantly higher TMB than those with early-stage (*P* = 0.03) (**Table 3**). No significant correlations were found between PD-L1 expression and tumor stage in patients with *HER2* mutations.

**Table 3:** Baseline characteristics by stage of NSCLC among matched patients with *HER2* mutations or amplification.

	*HER2* mutations			*HER2* amplification		
	I-III (*n* = 50)	IV (*n* = 50)	*Pval*	I-III (*n* = 21)	IV (*n* = 21)	*Pval*
Age, years			0.84			0.60
Mean (SD)	57.8 (9.2)	59.4 (9.2)		60.8 (8.0)	62.1 (9.3)	
Median (min-max)	56.5 (42-77)	59 (38-75)		61 (48-74)	64 (37-80)	
Gender, *n* (%)			0.84			0.75
Male	24 (48.0)	26 (52.0)		17 (81.0)	16 (76.2)	
Female	26 (52.0)	24 (48.0)		4 (19.0)	5 (23.8)	
Smoking, *n* (%)			0.6			1.00
Ever	10 (20.0)	14 (28.0)		11 (52.4)	10 (47.6)	
Never	17 (34.0)	17 (34.0)		5 (23.8)	5 (23.8)	
Unknown	23 (46.0)	19 (38.0)		5 (23.8)	6 (28.6)	
Histology, *n* (%)			0.24^a^			0.18^a^
Adenocarcinoma	46 (92.0)	48 (96.0)		12 (57.1)	17 (81.0)	
Squamous cell carcinoma	1 (2.0)	0		8 (38.1)	4 (19.0)	
Adenosquamous carcinoma	2 (4.0)	0		1 (4.8)	0	
Unknown	1 (2.0)	2 (4.0)		0	0	
TMB test, *n* (%)			0.03			0.62
Yes	47 (94.0)	47 (94.0)		20 (95.2)	20 (95.2)	
No	3 (6.0)	3 (6.0)		1 (4.8)	1 (4.8)	
Mean (SD)	3.9 (5.1)	6.3 (5.0)		9.2 (7.8)	8.1 (5.6)	
Median (min-max)	2.9 (0-26)	5.0 (1.0-28.8)		6.2 (1.0-27.8)	6.8 (1.0-20.2)	
PD-L1 test, *n* (%)			0.12			1.00
Yes	9 (18.0)	16 (32.0)		9 (42.9)	9 (42.9)	
No	41 (82.0)	34 (68.0)		12 (57.1)	12 (47.1)	
TPS<1%	3 (33.3)	11 (68.8)		6 (66.7)	6 (66.7)	
TPS≥1%	6 (66.7)	5 (31.2)		3 (33.3)	3 (33.3)	

a Comparison between adenocarcinoma and non-adenocarcinoma.

Across 62 patients with *HER2* amplification only, 21 (33.9%) and 41 (66.1%) patients were at early and late stage respectively. Patients with late-stage NSCLC had a higher proportion of adenocarcinoma than those with early stage (**Supplementary Table 3**). Similarly, a manual propensity-matched comparison was performed to compare the difference in genomic alterations between early- and late-stage NSCLC with *HER2* amplification only. We identified 21 out of the 41 patients from the late-stage group that matched with 21 patients with early-stage NSCLC, and their clinical characteristics are displayed in **Table 3**. No statistical differences were detected when comparing the frequency of co-alterations, TMB value, and PD-L1 expression level between the two matched groups. Therefore, molecular characteristics and PD-L1 expression of *HER2*-amplified NSCLC tumors were independent of tumor stages.

### 3.3 The efficacy of afatinib and pyrotinib in NSCLC patients with *HER2* alterations

To assess the efficacy of targeted therapy in NSCLC patients with *HER2* alterations, we retrospectively enrolled patients with *HER2* alterations who received anti-HER2 targeted therapy in our center. Twenty-one patients were eligible, and their clinical and molecular characteristics are presented in **Table 4**. The median age was 56 years (range, 39-76), and 52.4% (11/21) were female. Most patients had stage IV disease (81.0%, 17/21) and adenocarcinoma (95.2%, 20/21). *Via* genetic testing, most patients carried *HER2* exon 20 insertion (76.2%, 16/21), one carried *HER2* V659E mutation, one carried *HER2* S310X (exact sequence unknown) mutation, two carried *HER2* amplification only, and one carried *HER2* alteration with unclear sequence.

**Table 4:** Clinical and molecular features of patients with *HER2* alterations treated with anti-targeted therapy.

PatientID	Gender	Histology	Tumor stage	Age at diagnosis	Therapy line	anti-HER2 therapy	Best Response	PFS, m	Pre-tx *HER2* alteration	Pre-tx sample type	Post-tx sample type	Panel
P01	Male	Ad	IV	55	3	Pyrotinib	PR	6.8	Exon 20 ins (exact sequence unknown)	T	NA	–
P02	Male	Ad	III	52	2	Pyrotinib	PR	3.7	p.Y772_A775dup, amp	B	NA	–
P03	Female	Ad	IV	55	2	Pyrotinib	SD	2.4	p.G779_P780dup	T	NA	–
P04	Male	Ad	IV	61	2	Pyrotinib	PR	6.3 (ongoing)	Exact sequence unknown	–	NA	–
P05	Female	Ad	IV	56	3	Pyrotinib	SD	4	Exon 20 ins (exact sequence unknown)	H	NA	–
P06	Female	Ad	IV	50	3	Pyrotinib	SD	4.9	Exon 20 ins (exact sequence unknown)	B	NA	–
P07	Male	Ad	IV	63	2/3	Pyrotinib/Pyrotinib	SD/SD	3.5/2.8	p.Y772_A775dup	T	NA	–
P08	Female	Ad	IV	60	2/3	Pyrotinib/Pyrotinib	SD/SD	10.2/4.9	p.Y772_A775dup	T	NA	–
P09	Female	Ad	IV	76	1	Pyrotinib	SD	12.2	p.Y772_A775dup	–	NA	–
P10	Male	Ad	III	50	1	Pyrotinib	CR	33.3 (ongoing)	p.S310X (exact sequence unknown), amp	–	NA	–
P11	Male	Ad	I	71	1	Pyrotinib	SD	9.5	Amp	T	NA	–
P12	Male	Ad	IV	62	1	Pyrotinib	PD	2.2	p.Y772_A775dup	T	B	1021
P13	Male	Ad	IV	57	1	Afatinib	PR	4	p.Y772_A775dup	T	T	1021
P14	Female	Ad	–	56	3	Afatinib	SD	2	p.G778_P780dup	T	T	59
P15	Female	LCLC	IV	53	3	Afatinib	–	6	p.V659E	T	B	1021
P16	Female	Ad	IV	48	4	Pyrotinib	–	5	p.Y772_A775dup	C	C	1021
P17	Female	Ad	IV	62	2	Afatinib	PD	7.8	p.G778_P780dup	H	H	1021
P18	Male	Ad	IV	52	5	Afatinib	PD	1	Amp	T	B	59
P19	Male	Ad	IV	63	1	Afatinib	–	5	p.Y772_A775dup	B	B	1021
P20	Male	Ad	IV	39	2	Afatinib	PD	3	p.Y772_A775dup	T	B	1021
P21	Female	Ad	IV	60	4	Pyrotinib	–	14	p.Y772_A775dup	T	B	1,021

Ad, adenocarcinoma; LCLC, large cell lung cancer; T, tissue; H, hydrothorax; B, blood; C, cerebrospinal fluid; –, data not available; NA, not applicable.

**Figure 3:**
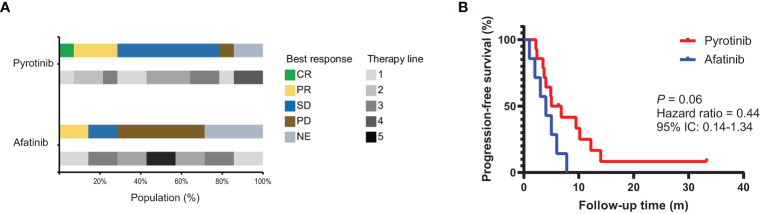
The efficacy of afatinib and pyrotinib in NSCLC patients with *HER2* alterations. **(A)** The proportion of best clinical response and therapy line for *HER2*-altered NSCLC patients treated with afatinib (*n* = 7) or pyrotinib (*n* = 14). **(B)** Kaplan–Meier estimates of progression-free survival of *HER2*-altered NSCLC patients treated with afatinib or pyrotinib.

Among these 21 patients, 14 (66.7%) patients received pyrotinib and 7 (33.3%) patients received afatinib. Two patients received pyrotinib as both second- and third-line treatment, of whom the clinical efficacy was only assessed on the second-line treatment. At the last follow-up date of October 2022, two patients were still on pyrotinib treatment and did not reach PFS, 18 patients developed disease progression, and one had died from the disease. The median line of afatinib and pyrotinib treatment was 2 (range, 1-5) and 2 (range, 1-4) respectively. The clinical response to targeted therapy was evaluated according to RECIST v1.1. As a result, pyrotinib yielded an ORR of 33.3% (4/12) and a DCR of 91.67% (11/12), and afatinib produced an ORR of 20% (1/5) and a DCR of 20% (1/5) (**Figure 3A**). The total mPFS was 5.0 months (95% CI, 3.8-10.1). Of note, patients who received pyrotinb treatment had a mPFS of 5.9 months (95% CI, 3.8-13.0), which is longer than those who received afatinib (4.0 months [95% CI, 1.9-6.3]), although statistical significance was not reached (*P* = 0.06) (**Figure 3B**). However, due to the limited number of patients enrolled in this analysis, this result is preliminary.

### 3.4 Potential resistance to afatinib and pyrotinib in NSCLC patients with *HER2* alterations

**Figure 4:**
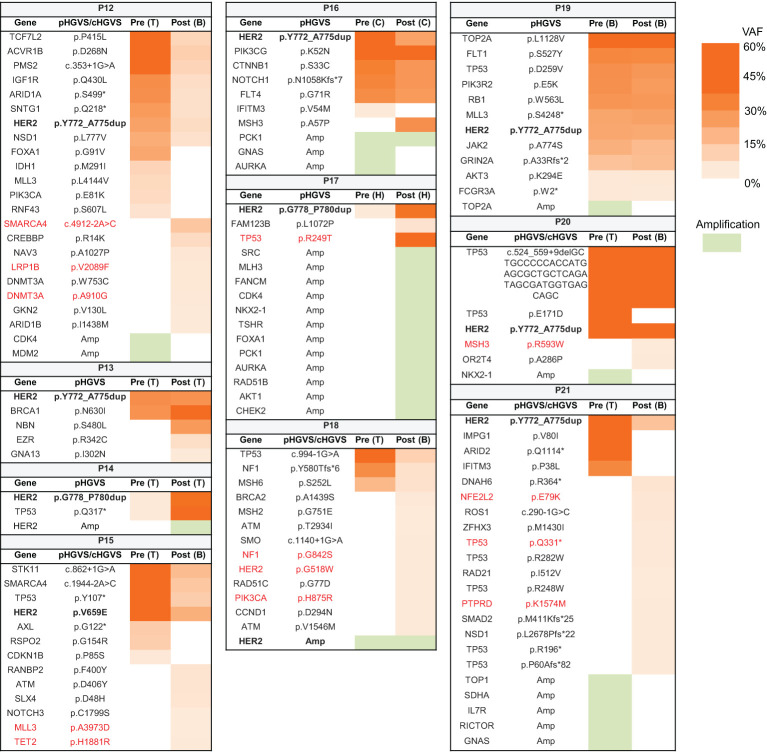
Potential resistance mechanism to afatinib and pyrotinib in lung cancer patients with *HER2* alterations. Heat map showing the variants and their allele frequencies in patients with *HER2* alterations before and after treatment. The acquired oncogenic mutations after treatment are marked with red color. B, blood; C, cerebrospinal fluid; H, hydrothorax; T, tissue; VAF, variant allele frequency.

Pre- and post-treatment genomic landscapes were dissected to uncover the potential resistance to afatinib and pyrotinib in ten eligible patients. Among them, nine patients carried *HER2* mutations, and one carried *HER2* amplification (**Figure 4**). All patients still harbored *HER2* alterations after afatinib or pyrotinib treatment. We first assessed the *HER2*-dependent resistance to afatinib or pyrotinib. For patient P14 harboring *HER2* G778_p780dup mutations and received afatinib as second-line treatment for 2 months, *HER2* amplification was identified at disease progression. For patient P18, carrying *HER2* amplification and received fifth-line afatinib treatment for one month, *HER2* G518W oncogenic mutation and *PIK3CA* mutation were found upon progression disease (PD) (**Figure 4**).

To explore *HER2*-independent resistance to afatinib and pyrotinib, we analyzed the acquired alterations from eight patients who did not have *HER2* secondary alterations at PD. Mutations reported by COSMIC datasets as oncogenic mutations were elaborately analyzed. Ten oncogenic mutations were identified in five patients. *TP53* participating in DNA damage repair (DDR) pathway, its oncogenic mutation was identified in two patients (P17 and P21) who received second-line afatinib and fourth-line pyrotinib respectively. Other alterations of DDR pathway mediated by *MSH2* oncogenic mutation was found in patient P20, who received second-line afatinib. Of note, two of these three patients harbored high genomic instability, with acquired 12 copy number alterations in P17 and acquired 13 mutations in P21, likely due to the deficiency of DDR pathway. *SMARCA4* gene, encoding a part of the SWI-SNF chromatin-remodeling complex, its loss of function mutation was detected in patient P12 treated with first-line pyrotinib, indicating that compromise of the SWI–SNF complex potentially confers resistance to pyrotinib. Additionally, mutations occurred in genes involved in epigenetic regulation (*LRP1B*, *DNMT3A*, *MLL3*, and *TET2*) were found in two patients (P12 and P15), suggesting epigenetic dysregulating might also play a role in resistance to HER2 targeted therapy.

## 4 Discussion

With growing evidence that anti-HER2 targeted therapy provides clinical efficacies in *HER2*-altered NSCLC, a comprehensive understanding of the clinical and molecular characteristics of *HER2*-altered NSCLC tumors is urgently needed. Our retrospective cohort of 176 treatment-naïve NSCLC patients with *HER2* alterations was elaborately analyzed to understand the molecular characteristics of *HER2*-altered NSCLC tumors. *HER2* lacks ligand binding domain and activates downstream signaling by binding to other ligand-bound epidermal growth factors to form a heterodimer. *HER2* oncogenic mutations are located in both TKD and non-TKD, which play roles in receptor dimerization. Our results showed that patients with oncogenic non-TKD mutations were older, and had a higher prevalence of *EGFR*, *KEAP1*, and *RBM10* mutations and *EGFR* amplification than those with oncogenic TKD mutation, indicating that *HER2* oncogenic non-TKD mutations exhibit weaker oncogenic features than *HER2* oncogenic TKD mutations. Previous studies have suggested that *HER2* mutations and amplification are distinct entities and therapeutic targets owing to no or low overlap (8, 24). In our study, 9.7% (17/176) patients had concurrent *HER2* mutation and amplification, which is consistent with the previous study (24). *HER2* oncogenic mutations are almost mutually exclusive with other driver mutations, indicating a powerful function in lung carcinogenesis. Patients with *HER2* amplification only had a higher prevalence of *EGFR* mutations and amplification, supporting that *HER2* amplification has a distinct entity and may be less relevant in lung carcinogenesis. Moreover, we found that patients with *HER2* amplification only had higher TMB than those with *HER2* mutations in early-stage disease. The elevated TMB in early-stage *HER2*-amplified NSCLC suggests a weaker oncogenic feature of *HER2* amplification as well. Furthermore, previous studies reported similar results that TMB is higher in NSCLC patients with *HER2* amplification than those with *HER2* mutations in mixed cohorts consisting of early and advanced disease, which is thought to be associated with the higher prevalence of male smokers in *HER2*-amplified group (25, 26). The comparable TMB between *HER2*-mutated and *HER2*-amplified NSCLC in late-stage disease was likely due to the increased tumor mutation load of late-stage *HER2*-mutated tumors, as indicated by our results that in *HER2*-mutated NSCLC, patients with late-stage disease had higher TMB than those with early-stage disease.

Co-alteration profiles of early- and late-stage NSCLC with *HER2* mutations or amplification only were studied. *TP53* mutation and high TMB are known prognostic factors of poor survival in NSCLC (27–29). Compared with *HER2*-mutant patients with early-stage NSCLC, those with late-stage NSCLC harbored higher frequency of *TP53* mutation and higher TMB. This result is congruent with the aggressive tumor biology of advanced NSCLC. However, the co-alteration profiles and TMB were similar in *HER2*-amplified patients with early- or late-stage NSCLC, providing evidence that *HER2* mutations and amplification may induce different lung carcinogenesis procedures.

Chemotherapy is the standard treatment for patients with *HER2*-mutant or -amplified NSCLC but showed unsatisfactory efficacies. Currently, several studies focused on the clinical response of HER2 TKIs in *HER2*-positive NSCLC, including afatinib, dacomitinib, neratinib, poziotinib and pyrotinib (11). Among them, afatinib has shown controversial clinical benefits results, with an ORR of 0%-33% and a mPFS of 2.76-3.7 months (11, 13–15). Our cohort enrolled seven patients received afatinib treatment, and only one with *HER2* Y772_A775dup achieved a partial response. The mPFS afatinib yielded was 4.0 months, which is comparable with previous reports (11, 13). Increasing evidences suggested pyrotinib had superior antitumor activity over afatinib with an ORR of 19.2-53.3% and a mPFS of 5.6-6.8 months (6, 11). Indeed, our retrospective study included 14 patients received pyrotinib and suggested an ORR of 33.3% and a mPFS of 5.9 months. The absence of statistical significance of mPFS between these two groups may be explained by the limited number of patients. In our cohort, pyrotinib produced a mPFS of 5.9 months, which is slightly shorter than Zhou C’s study but similar to Song Z’s study (7, 13). Differences in the sample size and between retrospective and prospective cohorts may lead to this discrepancy. Of great interest, patient P10 with IIIB adenocarcinoma received first-line pyrotinib treatment, and had a long-term complete remission of 33.3 months. This patient carried *HER2* S310X mutation, which is reminiscent of previous results that patients with non-exon 20 *HER2* mutations have superior clinical benefits from pyrotinib than those with exon 20 *HER2* mutations (6). However, due to the low incidence of non-exon 20 *HER2* mutations, the efficacy of pyrotinib in large cohorts with non-exon 20 *HER2* mutations requires further investigation. Additionally, trastuzumab deruxtecan, a novel HER2 antibody-drug conjugate, has shown promising therapeutic effects in *HER2*-mutant NSCLC with an ORR of 61.9% and a mPFS of 14.0 months in a phase II trial (30). We propose that pyrotinib and HER2-targeted antibody-drug conjugates may provide promising treatment options for patients with *HER2*-mutant NSCLC.

We investigated the potential resistance mechanism to afatinib and pyrotinib in 10 *HER2*-positive NSCLC by comparing pre- and post-treatment genomic profiles. *HER2* amplification was identified from one patient, consistent with previous reports that *HER2* amplification conferred resistance to anti-HER2 targeted therapy in *HER2*-mutant NSCLC (6, 31). In another patient, *HER2* G518W oncogenic mutation combined with *PIK3CA* mutation was identified at PD. Since a preclinical study suggested that dual PI3K/Akt/mTOR and HER2 blockade could conquer the resistance of anti-HER2 therapy (32), we propose that combing anti-HER2 agent with mTOR inhibitor might be a potential treatment option to reinforce the antitumor activities in this subset of patients. Three patients carried oncogenic mutations involved in the DDR pathway at PD. Since mutations in the DDR pathway were associated with improved survival for immune checkpoint inhibitors in NSCLC (33–35), a combination of pyrotinib and immune checkpoint inhibitors may vanquish the resistance. *SMARCA4* oncogenic mutation was identified in one patient who received pyrotinib. As an inhibitor of the pro-oncogenic transcriptional coactivators, *SMARCA4* is associated with increased metastatic incidence and leads to afatinib resistance in a patient with *EGFR*-mutant NSCLC (36, 37). We speculated that *SMRACA4* mutation might also play a role in HER2-TKI resistance. Additionally, mutations functioning in epigenetic regulating might also lead to HER2-TKI resistance. However, *in vivo*, *in vitro* and clinical evidences are needed to determine the biological impact of these mutations to anti-HER2 targeted therapy and to study the strategies to overcome these resistances.

This study has certain limitations. First, survival data were unavailable to analyze the prognostic value of *HER2* alteration. Second, comparison with afatinib and pyrotinib to *HER2*-mutant NSCLC should be validated in a larger cohort, especially a prospective cohort. Third, the number of patients used to evaluate the resistance mechanism to afatinib and pyrotinib was limited, and sample types before and after treatment were different. Since circulating tumor DNA is easier to obtain, it is recommended to use blood sample profiling to investigate resistance to anti-HER2 TKIs in a larger NSCLC population.

## 5 Conclusion

Our study displayed the molecular characteristics in treatment-naïve *HER2*-altered NSCLC. Compared with afatinib, pyrotinib showed better efficacy in patients with *HER2* alterations. Both *HER2*-dependent and -independent resistance mechanisms were found in patients treated with HER2 TKIs. Further larger cohorts, especially prospective cohorts, are warranted to validate the clinical activity and resistance mechanism of pyrotinib in NSCLC patients with *HER2* alterations.

## Data availability statement

The data presented in the study are deposited in the Genome Sequence Archive (Genomics, Proteomics & Bioinformatics 2021) in National Genomics Data Center (Nucleic Acids Res 2022), China National Center for Bioinformation / Beijing Institute of Genomics, Chinese Academy of Sciences that are publicly accessible at https://ngdc.cncb.ac.cn/gvm, accession number: GVM000466.

## Ethics statement

The studies involving human participants were reviewed and approved by the ethics board of Peking Union Medical College Hospital. Written informed consent for participation was not required for this study in accordance with the national legislation and the institutional requirements.

## Author contributions

YW, YX, and RC contributed to conception and design of the study. YW, YH, TL, and YX collected the data and organized the database. YX and YH performed data analysis and interpretation. YX and YH contributed to manuscript writing. All authors contributed to manuscript revision, read and approved the submitted version. All authors contributed to the article and approved the submitted version.
