# Supplementary material for: Genomic landscape and efficacy of HER2-targeted therapy in patients with HER2-mutant non-small cell lung cancer
Source: Front Oncol. 2023 Apr 3;13:1121708. doi: 10.3389/fonc.2023.1121708 (PMC10106648; doi:10.3389/fonc.2023.1121708)
Supplement: Supplementary file 1 [file Table_1.docx]

**Supplementary Table 1. List of target regions of 1,021 cancer-related genes.**

| **whole exons** |  |  |  |  |  |  |  |  |
| --- | --- | --- | --- | --- | --- | --- | --- | --- |
| *ABL1* | *ABL2* | *ACVR1B* | *AKT1* | *AKT2* | *AKT3* | *ALK* | *APC* | *AR* |
| *ARAF* | *ARID1A* | *ARID1B* | *ARID2* | *ASXL1* | *ATM* | *ATR* | *ATRX* | *AURKA* |
| *AURKB* | *AXIN1* | *AXIN2* | *AXL* | *B2M* | *BAP1* | *BARD1* | *BCL2* | *BCL2L1* |
| *BCOR* | *BLM* | *BMPR1A* | *BRAF* | *BRCA1* | *BRCA2* | *BRD4* | *BRIP1* | *BTK* |
| *C11orf30* | *CASP8* | *CBFB* | *CBL* | *CCND1* | *CCND2* | *CCND3* | *CCNE1* | *CD274* |
| *CDC73* | *CDH1* | *CDK12* | *CDK4* | *CDK6* | *CDK8* | *CDKN1A* | *CDKN1B* | *CDKN2A* |
| *CDKN2B* | *CDKN2C* | *CEBPA* | *CHEK1* | *CHEK2* | *CIC* | *CREBBP* | *CRKL* | *CSF1R* |
| *CTCF* | *CTNNA1* | *CTNNB1* | *CUL3* | *CYLD* | *DAXX* | *DDR1* | *DDR2* | *DICER1* |
| *DNMT3A* | *EGFR* | *ELAC2* | *EME2* | *EP300* | *EPAS1* | *EPCAM* | *EPHA2* | *EPHA3* |
| *EPHA5* | *EPHB2* | *EPHB6* | *ERBB2* | *ERBB3* | *ERBB4* | *ERCC1* | *ERCC3* | *ERG* |
| *ERRFI1* | *ESR1* | *EXT1* | *EXT2* | *EZH2* | *FAM123B* | *FAM175A* | *FANCA* | *FANCC* |
| *FANCD2* | *FANCG* | *FANCM* | *FAS* | *FAT1* | *FAT2* | *FBXW7* | *FCGR2A* | *FCGR3A* |
| *FGFR1* | *FGFR2* | *FGFR3* | *FGFR4* | *FH* | *FLCN* | *FLT1* | *FLT3* | *FLT4* |
| *FOXA1* | *FOXL2* | *FOXP1* | *FUBP1* | *GAB2* | *GALNT12* | *GATA3* | *GNA11* | *GNAQ* |
| *GNAS* | *GRIN2A* | *HDAC1* | *HDAC4* | *HGF* | *HNF1A* | *HOXB13* | *HRAS* | *HSP90AA1* |
| *IDH1* | *IDH2* | *IFNG* | *IFNGR1* | *IGF1R* | *IL7R* | *INPP4B* | *IRF2* | *IRS2* |
| *JAK1* | *JAK2* | *JAK3* | *KDM5A* | *KDM5C* | *KDM6A* | *KDR* | *KEAP1* | *KIT* |
| *KRAS* | *LRP1B* | *MAP2K1* | *MAP2K2* | *MAP2K4* | *MAP3K1* | *MAPK1* | *MAX* | *MCL1* |
| *MDM2* | *MDM4* | *MED12* | *MEN1* | *MET* | *MITF* | *MLH1* | *MLH3* | *MLL* |
| *MLL2* | *MLL3* | *MPL* | *MRE11A* | *MS4A1* | *MSH2* | *MSH3* | *MSH6* | *MTOR* |
| *MUTYH* | *MYC* | *MYCL1* | *MYCN* | *MYD88* | *NBN* | *NCOR1* | *NDUFA13* | *NF1* |
| *NF2* | *NOTCH1* | *NOTCH2* | *NOTCH3* | *NOTCH4* | *NPM1* | *NRAS* | *NSD1* | *NTHL1* |
| *NTRK1* | *NTRK3* | *PALB2* | *PAX5* | *PBRM1* | *PCK1* | *PDCD1LG2* | *PDGFRA* | *PDGFRB* |
| *PDK1* | *PHF6* | *PIK3CA* | *PIK3CB* | *PIK3CG* | *PIK3R1* | *PIK3R2* | *PMS1* | *PMS2* |
| *POLD1* | *POLE* | *POT1* | *PPM1D* | *PRKAR1A* | *PTCH1* | *PTCH2* | *PTEN* | *PTPN11* |
| *RAD50* | *RAD51* | *RAD51B* | *RAD51C* | *RAD51D* | *RAF1* | *RARA* | *RB1* | *RBM10* |
| *RET* | *RHEB* | *RHOA* | *RICTOR* | *RINT1* | *RNASEL* | *RNF43* | *ROS1* | *RPS6KB1* |
| *RUNX1* | *SDHA* | *SDHAF2* | *SDHB* | *SDHC* | *SDHD* | *SERPINB3* | *SERPINB4* | *SETD2* |
| *SLX4* | *SMAD2* | *SMAD4* | *SMARCA4* | *SMARCB1* | *SMARCE1* | *SMO* | *SOX2* | *SOX9* |
| *SRC* | *STAG2* | *STAT3* | *STK11* | *SUFU* | *SYK* | *TBX3* | *TCF7L2* | *TET2* |
| *TGFBR2* | *TMEM127* | *TMPRSS2* | *TNFAIP3* | *TOP1* | *TOP2A* | *TP53* | *TP73* | *TSC1* |
| *TSC2* | *VEGFA* | *VHL* | *WT1* | *XPO1* | *XRCC2* | *XRCC3* | *ZFHX3* | *ZMAT3* |
|  |  |  |  |  |  |  |  |  |
| **intron, promoter, fusion points/breakpoints** |  |  |  |  |  |  |  |  |
| *ALK* | *BCL2L11* | *BRAF* | *BRCA1* | *BRD4* | *CD74* | *EGFR* | *EML4* | *ERG* |
| *ETV6* | *EZR* | *FGFR1* | *FGFR2* | *FGFR3* | *KIF5B* | *KIT* | *MAML2* | *MET* |
| *MSH2* | *MYC* | *MYCL1* | *NCOA4* | *NOTCH2* | *NTRK1* | *NTRK2* | *NTRK3* | *PDGFRA* |
| *PMS2* | *PPARG* | *RAF1* | *RET* | *ROS1* | *RSPO2* | *SLC34A2* | *TERT* | *TFE3* |
| *TMPRSS2* | *TPM3* |  |  |  |  |  |  |  |
|  |  |  |  |  |  |  |  |  |
| **partial exons** |  |  |  |  |  |  |  |  |
| *ABCA13* | *ABCB1* | *ABCC1* | *ABCC11* | *ABCC2* | *ABCG2* | *ACACA* | *ACIN1* | *ACTB* |
| *ACTG1* | *ACTG2* | *ACVR2A* | *ACVRL1* | *ADAM29* | *ADAMTS5* | *ADCY1* | *AFF1* | *AFF2* |
| *AFF3* | *AFF4* | *AHNAK* | *AKAP9* | *ALB* | *AMOT* | *ANGPT1* | *ANK3* | *ANKRD27* |
| *ANKRD30A* | *ANKRD30B* | *ANKRD36B* | *APEX1* | *APOBEC3B* | *ARAP3* | *ARFGEF1* | *ARFGEF2* | *ARHGAP26* |
| *ARHGAP29* | *ARHGAP35* | *ARID4B* | *ARNT* | *ASCL4* | *ASH1L* | *ASMTL* | *ASPM* | *ASTN1* |
| *ASXL2* | *ATIC* | *ATP12A* | *ATP11B* | *ATP1A1* | *ATP2B3* | *BAZ2B* | *BBS9* | *BCAS1* |
| *BCL11A* | *BCL11B* | *BCL2A1* | *BCL2L11* | *BCL3* | *BCL9* | *BCLAF1* | *BCORL1* | *BCR* |
| *BIRC2* | *BIRC3* | *BMPR2* | *BNC2* | *BPTF* | *BRD2* | *BRD3* | *BRSK1* | *BRWD1* |
| *BTLA* | *BUB1* | *C15orf23* | *C15orf55* | *C1QA* | *C1S* | *C3orf70* | *C7orf53* | *C8orf34* |
| *CACNA1D* | *CACNA1E* | *CADM2* | *CAMTA1* | *CAPN7* | *CARD11* | *CASP1* | *CASQ2* | *CBLB* |
| *CBR1* | *CBR3* | *CCDC168* | *CCNA1* | *CCNB3* | *CCT3* | *CCT5* | *CCT6B* | *CD22* |
| *CD33* | *CD5L* | *CDA* | *CDH11* | *CDH18* | *CDH23* | *CDK13* | *CHD1* | *CHD1L* |
| *CHD3* | *CHD4* | *CHD6* | *CHD8* | *CHD9* | *CHFR* | *CHI3L1* | *CHN1* | *CIITA* |
| *CKS1B* | *CLCC1* | *CLDN18* | *CLP1* | *CLSPN* | *CLTC* | *CNOT3* | *CNOT4* | *CNTN1* |
| *CNTN5* | *CNTNAP1* | *CNTNAP5* | *COL1A1* | *COL2A1* | *COL5A1* | *COL5A2* | *COL5A3* | *COPS2* |
| *CPS1* | *CREB3L1* | *CRIPAK* | *CRLF2* | *CRNKL1* | *CRTC1* | *CRYBG3* | *CSF1* | *CSF3R* |
| *CSMD1* | *CSMD3* | *CSNK1A1* | *CSNK1G3* | *CSNK2A1* | *CTLA4* | *CTNNA2* | *CTNND1* | *CUX1* |
| *CYBA* | *CYP19A1* | *CYP1B1* | *CYP1A1* | *CYP2A13* | *CYP2C19* | *CYP2C8* | *CYP2D6* | *CYP3A4* |
| *CYP3A5* | *DCC* | *DDX3X* | *DDX5* | *DEK* | *DHX35* | *DHX9* | *DIAPH1* | *DIS3L2* |
| *DLC1* | *DMD* | *DNAH6* | *DNAJC11* | *DNM2* | *DNMT1* | *DOCK2* | *DOCK7* | *DOT1L* |
| *DPYD* | *DRGX* | *DTX1* | *DUSP22* | *DYSF* | *EBF1* | *ECT2L* | *EEF1A1* | *EGR3* |
| *EIF2AK3* | *EIF2C3* | *EIF3A* | *EIF4G3* | *ELF1* | *ELF3* | *ELF4* | *ELL* | *ELMO1* |
| *ELN* | *EMID2* | *EPC1* | *EPHA1* | *EPHA4* | *EPHA7* | *EPHB1* | *EPHB4* | *EPOR* |
| *EPPK1* | *EPS15* | *ERBB2IP* | *ERCC2* | *ESR2* | *ETS1* | *ETV1* | *ETV5* | *ETV6* |
| *EWSR1* | *EZR* | *F8* | *FAM131B* | *FAM135B* | *FAM157B* | *FAM22A* | *FAM46C* | *FAM5C* |
| *FAP* | *FASLG* | *FAT3* | *FAT4* | *FCGR1A* | *FCGR2B* | *FCRL4* | *FGF10* | *FGF14* |
| *FGF23* | *FGF3* | *FGF4* | *FGF6* | *FKBP5* | *FLG* | *FLI1* | *FLNC* | *FMN2* |
| *FMR1* | *FN1* | *FNDC4* | *FOXA2* | *FOXO3* | *FOXQ1* | *FRG1* | *FRMPD4* | *FUS* |
| *FXR1* | *FYN* | *FZD1* | *G3BP1* | *G3BP2* | *GABRA6* | *GATA2* | *GFRAL* | *GIGYF1* |
| *GKN2* | *GLB1L3* | *GLI1* | *GLI2* | *GLI3* | *GMPS* | *GNA12* | *GNA13* | *GNG2* |
| *GPC3* | *GPR124* | *GPX1* | *GRB7* | *GRM3* | *GSK3B* | *GSTM5* | *GSTP1* | *GUSB* |
| *H3F3A* | *H3F3C* | *HCLS1* | *HCN1* | *HDAC9* | *HECW1* | *HERC2* | *HEY1* | *HIP1* |
| *HIST1H1C* | *HIST1H1D* | *HIST1H1E* | *HIST1H2AC* | *HIST1H2AG* | *HIST1H2AL* | *HIST1H2AM* | *HIST1H2BC* | *HIST1H2BD* |
| *HIST1H2BJ* | *HIST1H2BK* | *HIST1H2BO* | *HIST1H3B* | *HIST1H4I* | *HLF* | *HMCN1* | *HNRPDL* | *HOXA11* |
| *HOXA13* | *HOXA3* | *HOXA9* | *HOXC13* | *HOXD11* | *HOXD13* | *HSD3B1* | *HSD3B2* | *HSP90AB1* |
| *HSPA8* | *HSPD1* | *HSPH1* | *ICK* | *IFITM1* | *IFITM3* | *IGF2* | *IGF2R* | *IGLL5* |
| *IGSF10* | *IKBKE* | *IKZF1* | *IKZF2* | *IKZF3* | *IL1RAPL1* | *IL21R* | *IL6* | *IL6ST* |
| *IMPG1* | *ING1* | *INHBA* | *INPP4A* | *INPP5D* | *INPPL1* | *IRF4* | *IRF6* | *ITGB3* |
| *ITK* | *ITSN1* | *JARID2* | *KALRN* | *KAT6A* | *KAT6B* | *KCNJ5* | *KCNQ2* | *KDM2B* |
| *KDM3B* | *KEL* | *KIF5B* | *KLB* | *KLF4* | *KLHL6* | *KLK1* | *KRTAP5-5* | *L3MBTL1* |
| *LAMA2* | *LCP1* | *LEF1* | *LGALS8* | *LIFR* | *LPHN2* | *LPP* | *LRP2* | *LRP4* |
| *LRP5* | *LRP6* | *LRRC7* | *LRRK2* | *LYN* | *LZTS1* | *MACF1* | *MAD1L1* | *MAGI2* |
| *MAGOH* | *MAML2* | *MAML3* | *MAP3K13* | *MAPK3* | *MCC* | *MCM3* | *MDH2* | *MECOM* |
| *MEF2C* | *MGA* | *MIB1* | *MIOS* | *MKI67* | *MKL1* | *MLL4* | *MLLT3* | *MLLT6* |
| *MMP2* | *MMP11* | *MN1* | *MNDA* | *MNX1* | *MPO* | *MSH4* | *MSN* | *MSR1* |
| *MTHFR* | *MTRR* | *MUC5B* | *MYB* | *MYBL2* | *MYH10* | *MYH11* | *MYH14* | *MYH9* |
| *MYO3A* | *NAP1L1* | *NAV3* | *NBPF1* | *NCAM2* | *NCF2* | *NCF4* | *NCK1* | *NCOA2* |
| *NCOR2* | *NCSTN* | *NDRG1* | *NEB* | *NFATC4* | *NFE2L2* | *NFE2L3* | *NIN* | *NKX3-1* |
| *NLRC3* | *NOD1* | *NOS3* | *NQO1* | *NR1I2* | *NR2F2* | *NR4A2* | *NRP2* | *NRXN1* |
| *NTM* | *NTRK2* | *NUMA1* | *NUP107* | *NUP210* | *NUP98* | *OBSCN* | *OGDH* | *OMD* |
| *OPCML* | *OR11G2* | *OR2T4* | *OR4A15* | *OR4C6* | *OR5L2* | *OR6F1* | *P2RY8* | *P4HB* |
| *PABPC1* | *PABPC3* | *PAG1* | *PAK1* | *PAK3* | *PARK2* | *PARP1* | *PASK* | *PAX3* |
| *PAX7* | *PBX1* | *PC* | *PCDH18* | *PCLO* | *PCSK6* | *PCSK7* | *PDCD1* | *PDCD11* |
| *PDE4DIP* | *PDGFB* | *PDILT* | *PER1* | *PGR* | *PHF1* | *PIK3C2A* | *PIK3C2B* | *PIK3C2G* |
| *PIK3R3* | *PIP5K1A* | *PKD1L2* | *PKHD1* | *PLAC8* | *PLAG1* | *PLCB1* | *PLCG1* | *PLCG2* |
| *PLK1* | *PLXNA1* | *PLXNB2* | *POLQ* | *POLR2B* | *POM121* | *POM121L12* | *POTEG* | *POU2AF1* |
| *PPP1R17* | *PPP2R1A* | *PPP6C* | *PRAM1* | *PRDM1* | *PRDM16* | *PREX2* | *PRF1* | *PRKAA1* |
| *PRKCB* | *PRKCI* | *PRKDC* | *PRRX1* | *PRX* | *PSG2* | *PSIP1* | *PSMB1* | *PSMB5* |
| *PTGS1* | *PTGS2* | *PTK2* | *PTPN13* | *PTPN2* | *PTPRB* | *PTPRD* | *PTPRF* | *PTPRJ* |
| *PTPRK* | *PTPRO* | *PTPRT* | *PTPRU* | *RAB35* | *RAC1* | *RAC2* | *RAD21* | *RAD54B* |
| *RANBP2* | *RASA1* | *RASGRP1* | *RBL1* | *RECQL4* | *REL* | *RELN* | *RFC1* | *RGS3* |
| *RHOH* | *RHOT1* | *RIT1* | *RNF213* | *ROBO1* | *ROBO2* | *ROBO3* | *ROCK1* | *RPGR* |
| *RPL22* | *RPTOR* | *RSPO2* | *RSPO3* | *RUNX1T1* | *RUNX2* | *RXRA* | *RYR1* | *RYR2* |
| *SBDS* | *SCUBE2* | *SEC31A* | *SEMA3A* | *SEMA3E* | *SEMA6A* | *SERP2* | *SERPINA7* | *SETBP1* |
| *SETDB1* | *SF1* | *SF3A1* | *SF3A3* | *SF3B1* | *SFPQ* | *SGCZ* | *SH3PXD2A* | *SHH* |
| *SI* | *SIN3A* | *SLC16A1* | *SLC1A2* | *SLC22A16* | *SLC22A18* | *SLC22A2* | *SLC22A3* | *SLCO1B3* |
| *SLIT1* | *SLIT2* | *SMAD3* | *SMC1A* | *SMC1B* | *SMURF2* | *SNCAIP* | *SNTG1* | *SNX29* |
| *SOD2* | *SOS1* | *SOX10* | *SOX17* | *SPEN* | *SPOP* | *SPRR3* | *SPSB4* | *SPTA1* |
| *SRD5A2* | *SRGAP1* | *SRGAP3* | *SRSF2* | *SRSF7* | *SSX1* | *STAG1* | *STAT1* | *STAT5A* |
| *SUCLG1* | *SUCLG2* | *SULT1A1* | *SUZ12* | *SVEP1* | *SYNCRIP* | *SYNE1* | *TAF1* | *TAF15* |
| *TAF1L* | *TAL1* | *TBL1XR1* | *TBX15* | *TBX22* | *TCEB1* | *TCERG1* | *TCF12* | *TCF3* |
| *TCF4* | *TCL1A* | *TCP11* | *TEC* | *TENM3* | *TERT* | *TFDP1* | *TFDP2* | *TFE3* |
| *TGFBR1* | *TGFBR3* | *TGM2* | *THBS1* | *THBS2* | *THRAP3* | *TJP1* | *TLE1* | *TLL2* |
| *TLR4* | *TLX3* | *TMEM132D* | *TNN* | *TNPO1* | *TOP2B* | *TP53BP1* | *TP63* | *TPM3* |
| *TPR* | *TRAF5* | *TRERF1* | *TRIM24* | *TRIM58* | *TRIO* | *TRPC5* | *TRRAP* | *TSHR* |
| *TSHZ2* | *TSHZ3* | *TTF1* | *TTL* | *TUBA3C* | *TUBB3* | *TUSC3* | *TXNIP* | *TYMS* |
| *TYR* | *TYRP1* | *U2AF1* | *UBE2D2* | *UBR5* | *UGT1A1* | *UMPS* | *UPF3B* | *USH2A* |
| *USP6* | *USP8* | *VDAC2* | *VEZF1* | *VIM* | *WASF3* | *WDR90* | *WDTC1* | *WHSC1* |
| *WHSC1L1* | *WIPF1* | *WNK1* | *WNT5A* | *WSCD2* | *WWOX* | *WWP1* | *WWP2* | *XBP1* |
| *XPC* | *XRCC1* | *YBX1* | *YY1AP1* | *ZBTB16* | *ZC3H11A* | *ZFP36L1* | *ZFP36L2* | *ZFPM2* |
| *ZIC3* | *ZNF217* | *ZNF384* | *ZNF521* | *ZNF638* | *ZNF750* | *ZNF804B* | *ZNF814* |  |
|  |  |  |  |  |  |  |  |  |
| **germline mutation** |  |  |  |  |  |  |  |  |
| *ATM* | *BRCA1* | *BRCA2* | *MLH1* | *MLH3* | *MSH2* |  |  |  |
| *MSH3* | *MSH6* | *PALB2* | *PMS1* | *PMS2* |  |  |  |  |

**Supplementary Table 2. List of target regions of 59 cancer-related genes.**

| **whole exons** |  |  |  |  |  |
| --- | --- | --- | --- | --- | --- |
| *ALK* | *ATM* | *BRAF* | *BRCA1* | *BRCA2* | *CCND1* |
| *CDK4* | *CDK6* | *CDKN2A* | *DDR2* | *EGFR* | *ERBB2* |
| *FBXW7* | *FGFR1* | *KIT* | *KRAS* | *MAP2K1* | *MET* |
| *MLH1* | *MSH2* | *MSH6* | *NF1* | *NRAS* | *NTRK1* |
| *PIK3CA* | *PMS2* | *PTCH1* | *PTEN* | *RB1* | *RET* |
| *ROS1* | *SMARCA4* | *SMO* | *STK11* | *TP53* | *TSC1* |
| *TSC2* |  |  |  |  |  |
|  |  |  |  |  |  |
| **partial exons** |  |  |  |  |  |
| *AKT1* | *APC* | *AR* | *CTNNB1* | *ESR1* | *FGFR2* |
| *FGFR3* | *FLT3* | *HRAS* | *IDH1* | *IDH2* | *JAK2* |
| *MAP2K2* | *MTOR* | *PDGFRA* | *PTPN11* | *RAF1* | *SRC* |
| *VHL* |  |  |  |  |  |
|  |  |  |  |  |  |
| **fusion points/breakpoints** |  |  |  |  |  |
| *ALK* | *FGFR3* | *NTRK1* | *PDGFRA* | *RET* | *ROS1* |

**Supplementary Table 3. Baseline characteristics by stage of NSCLC among patients with *HER2* mutations or amplification.**

|  | ***HER2* mutations** | |  | ***HER2* amplification** | |  |
| --- | --- | --- | --- | --- | --- | --- |
|  | **I-III (*n* = 60)** | **IV (*n* = 54)** | ***Pval*** | **I-III (*n* = 21)** | **IV (*n* = 41）** | ***Pval*** |
| **Age, years** |  |  | 0.002 |  |  | 0.58 |
| Mean (SD) | 54.2 (12.5) | 59.6 (9.6) |  | 60.8 (8.0) | 62.1 (9.3) |  |
| Median (min-max) | 55 (25-77) | 60 (38-76) |  | 61 (48-74) | 64 (37-80) |  |
| **Gender, *n* (%)** |  |  | 0.45 |  |  | 0.75 |
| Male | 25 (41.7) | 27 (50.0) |  | 17 (81.0) | 31 (75.6) |  |
| Female | 35 (58.3) | 27 (50.0) |  | 4 (19.0) | 10 (24.4) |  |
| **Smoking, *n* (%)** |  |  | 0.21 |  |  | 0.53 |
| Ever | 10 (16.7) | 15 (27.8) |  | 11 (52.4) | 17 (41.5) |  |
| Never | 25 (41.7) | 18 (33.3) |  | 5 (23.8) | 14 (34.1) |  |
| Unknown | 25 (41.7) | 21 (38.9) |  | 5 (23.8) | 10 (24.4) |  |
| **Histology, *n* (%)** |  |  | 0.25^a^ |  |  | 0.009^a^ |
| Adenocarcinoma | 56 (93.3) | 52 (96.3) |  | 12 (57.1) | 32 (78.0) |  |
| Squamous cell carcinoma | 1 (1.7) | 0 |  | 8 (38.1) | 4 (9.8) |  |
| Adenosquamous carcinoma | 2 (3.3) | 0 |  | 1 (4.8) | 0 |  |
| Unknown | 1 (1.7) | 2 (3.7) |  | 0 | 5 (12.2) |  |

a Comparison between adenocarcinoma and non-adenocarcinoma
